# Identification of the Temperature Dependence of the Thermal Expansion Coefficient of Polymers

**DOI:** 10.3390/polym13183035

**Published:** 2021-09-08

**Authors:** Igor N. Shardakov, Aleksandr N. Trufanov

**Affiliations:** 1 Institute of Continuous Media Mechanics, Ural Branch of Russian Academy of Sciences, 614013 Perm, Russia; shardakov@icmm.ru; 2 Department of Computational Mathematics, Mechanics and Biomechanics, Perm National Research Polytechnic University, 614990 Perm, Russia

**Keywords:** thermal properties, coefficient of thermal expansion, glass transition, thermal expansion behavior, polymer film sample, thermal cycles

## Abstract

In this paper, we proposed an approach to study the strain response of polymer film samples under various temperature effects and note their corresponding effects. The advantages of the developed approach are determined by the fact that thin films of material are used as samples where it is possible to generate a sufficiently uniform temperature field in a wide range of temperature change rates. A dynamic mechanical analyzer was used for the experimental implementation of the above approach for two UV-curable polymers and one type of epoxy resin. Experimental results have shown that the thermal expansion coefficients for these polymers depend significantly not only on the temperature but also on its change rate. The strain response of the polymer to heating and cooling, with the same absolute values of the rate of temperature change, differs significantly, and this dissimilarity becomes stronger with its increasing. The results of thermomechanical experiments for massive samples on traditional dilatometer are shown to compare with the results for film samples. The discovered dependences of the temperature expansion coefficient on the temperature and its change rate can be used for mathematical modeling of thermomechanical processes arising during the operation of products made of polymers.

## 1. Introduction

Structural polymeric materials are widely used in various fields [1,2,3]. Modern approaches to designing the thermomechanical behavior of products made of them require the completeness of information about the dependence of physical and mechanical properties of materials on the temperature. It is known that temperature changes significantly affect the thermomechanical properties of polymers [4,5]. At the same time, when thermal relaxation transitions take place, some parameters can change by times and even by orders of magnitude [6,7]. Thus, the development of approaches that allow establishing thermomechanical properties of polymers in a wide range of temperatures is relevant. An important place among these properties is occupied by the coefficient of thermal expansion (CTE).

Several works are devoted to the study of dependence of CTE on the parameters characterizing the temperature effect. In the article of A.I. Slutsker et al. [8], the temperature dependence of thermal expansion of polyvinyl acetate (PVA) in the region of relaxation transition at small harmonic temperature fluctuations relative to basic values was studied. The authors found that in the region of glass transition, a phase shift is observed between the expansion of the sample and the temperature, the magnitude of which depends on the frequency of temperature fluctuations. In order to explain the observed effect, the authors assumed that the temperature strain is determined not only by anharmonic vibrations of the atoms but also by the kinetics of conformational transitions. In the monograph of R. Houwink and A. Staverman [9], the dependences of the specific volume on temperature are plotted based on the results of experiments on samples of amorphous polymer, polymethylmethacrylate, are summarized. The glass transition temperature and the specific volume were found to depend on the cooling rate. R.S. Spencer [10] studied the effect of cooling rate on the specific volume of polystyrene and obtained experimental data, which were used to construct analytical dependences of the specific volume on temperature and the rate of its change. In the work of B.Y. Teitelbaum [11], it is also noted that the change of the specific volume in the region of glass transition temperatures depends on the rate of temperature change. It follows from the results of these papers that the temperature expansion of polymers depends not only on temperature but also on its time derivative. J.M. Hutchinson comes to similar conclusions in his theoretical papers [12,13], studying the dependence of temperature expansion of glasses on the history of temperature exposure.

The relevance of determining the functional dependencies of CTE on temperature parameters is further related to the fact that reference data and manufacturers’ specifications do not allow for determining the dependence of CTE on temperature and its rate of change. For example, the manufacturer’s data [14,15] for DeSolite polymers are given in Table 1.

The information in Table 1 rather crudely determines the temperature dependence of CTE on temperature, whereas the influence of the rate of temperature change is not considered at all. This circumstance does not allow the thermomechanical behavior of structures made of these polymers to be described correctly.

To summarize the above, it can be stated that the temperature dependence of the thermal expansion coefficient of polymers is a function not only of temperature but also of the rate of its change, which is especially evident in the intervals involving thermal relaxation transitions in the material. The presented paper considers the development of methods for identifying and verifying of the temperature dependence of the CTE in a wide temperature range, including the relaxation transition. The basic approaches have been tested on film samples of UV-curable polymers and epoxy resin.

## 2. Materials and Methods

When developing the methodology of the experiment for determining the dependence of CTE on temperature and its first time derivative in a wide temperature range, including the relaxation transition, we proceeded from the following assumptions. The methodology should make it possible to obtain data for polymers in the glassy and highly elastic states in the temperature range from −100 °C to +150 °C at varying rates of temperature change. 

The temperature field is also assumed to be sufficiently uniform over the sample volume during the experiment. Under conditions of varying temperature, this exact requirement is the main obstacle to the use of traditional dilatometers. The samples used in these measurements have a massive volumetric shape, which severely limits the permissible interval of the rate of temperature change. To overcome this difficulty, we used polymer film samples in our research. This shape of the samples significantly expanded the allowable interval of temperature change rate. The methods of laser interferometry [16], neutron reflectometry [17], spectral ellipsometry [18], and x-ray reflectometry [19] can be used to study the strain response of film samples to temperature and mechanical impact, which have high measurement accuracy and allow dealing with thin films. However, it should be noted that in all these approaches, difficulties lie in the ability to control the rate of temperature change and a narrow range of its permissible values.

Within the framework of the presented paper, the most rational approach for measuring temperature strains of film samples was the use of a dynamic mechanical analyzer (DMA). Such devices have several significant advantages; they include a thermal chamber, which allows setting the temperature in a wide range and regulating the rate of its change—the precise control of the applied force and measured displacements are provided. There is appropriate tooling, which allows working with film samples. A necessary condition for the correctness of results of measurement of material response to external temperature influence is uniformity of temperature field over the sample volume during the whole experiment. According to H.S. Carslaw and J.C. Jaeger [20], the uniformity of the temperature field can be characterized by the coefficient of non-uniformity ψ, equal to the ratio of the temperature in the middle of the sample to the temperature on its surface. For a uniform field, this coefficient is close to one.

We should highlight that the film shape of the samples provides a high degree of temperature field uniformity over the sample volume (ψ ≈ 1) at different speeds of external temperature influence. This property of film samples enables correct investigating of the dependence of CTE on the temperature field change rate. The range of admissible rates of temperature change for the study significantly decreases as the sample thickness increases because the coefficient ψ decreases in this case.

The advantage of the proposed approach is that it is possible to collect data on the same sample both to analyze thermal expansion and to determine viscoelastic characteristics.

Experimental studies in this paper were performed using a TA Instruments Q800 dynamic mechanical analyzer (TA Instruments Inc., New Castle, DE, USA) with a GCA liquid nitrogen cooling system, which allows the properties of polymers to be investigated in the temperature range from −150 to 600 °C. The manufacturer’s declared accuracy of displacement measurement is 1 nm, the accuracy of force measurement is 10 μN.

**Figure 1:**
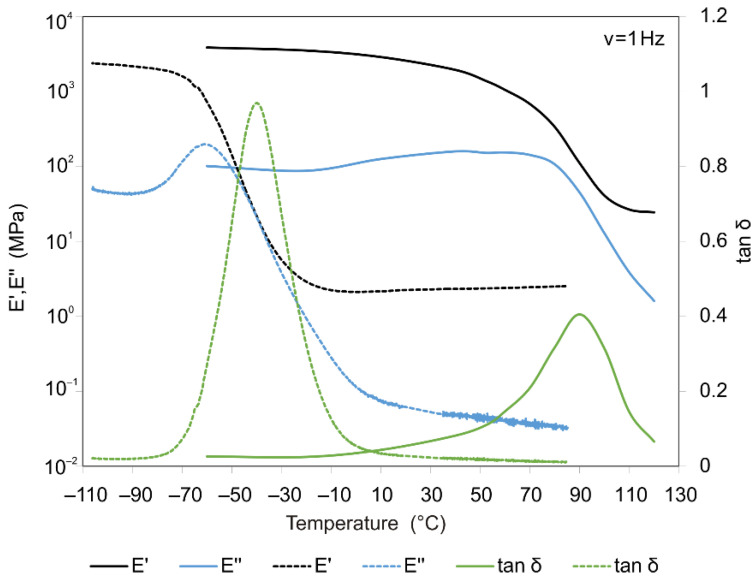
Results of dynamo-mechanical analysis of DeSolite 3471-1-152A (dashed line) and DeSolite DS-2015 (solid line) polymers, frequency 1 Hz, heating rate 2 °C/min.

Two UV-curable polymers from DSM Desotech (Elgin, IL, USA), produced under the DeSolite trademark, were investigated in this work. The first polymer was DeSolite 3471-1-152A and the second was DeSolite DS-2015. Film samples had the following dimensions: length 13–19 mm, width 6.2 mm, thickness 0.04–0.25 mm. At the first stage of the study, thermomechanical experiments were carried out for these polymers to determine the components of the complex module: the real part E′ (storage modulus) and the imaginary portion E″ (loss modulus). The results of these experiments made it possible to determine the loss factor tan(δ) and to estimate the temperature intervals in which the relaxation transitions are implemented. Figure 1 illustrates the obtained data. 

The next stage of the experimental studies is devoted to the main goal of this investigation—the determination of the dependence of CTE on temperature and its first time derivative in a wide temperature range, including the relaxation transition. A constant tensile force was applied to ensure that the shape of the film sample remains unchanged during the experiment, the value of which was chosen as minimally possible in the range from 0.001 to 0.005 N. At the same time, the additional strain caused by such tension was found not to exceed 5%.

The measurements were according to the following algorithm: 

(1) Immediately before starting the experiment, the samples were heated and held for up to 30 min at 130 °C, which is higher than the glass transition temperature (for DeSolite DS-2015 polymer) to remove the possible residual stresses and other effects related to the history of their manufacturing and storage.

(2) The sample was then slowly cooled down to the upper boundary of the temperature range under study. 

(3) The sample with applied tensile force was kept at the temperature corresponding to the upper boundary of the range under study. This provided full implementation of relaxation processes in the material. 

(4) Then, the sample was cooled down at a constant rate to the lower boundary of the temperature range, kept until the strain stabilized, and then heated at the same rate to the upper boundary of the range. At the end of heating, the samples were kept at a constant temperature again until the strain stabilized. 

**Figure 2:**
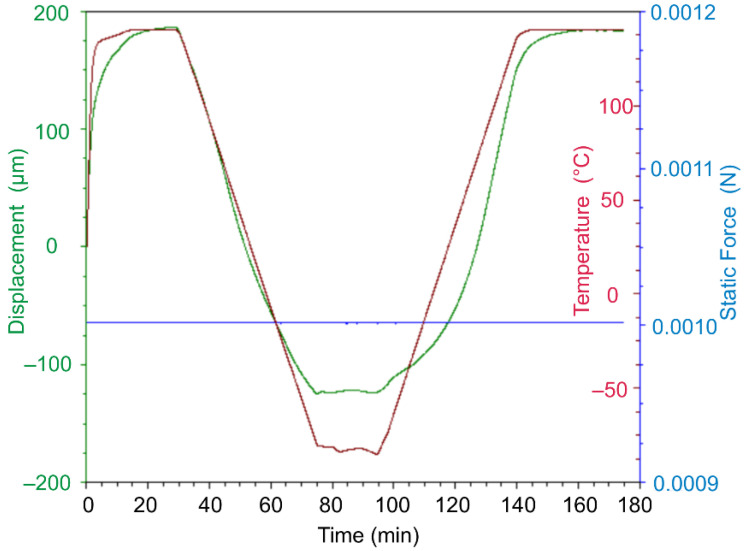
Characteristic time dependencies of the parameters recorded during the experiment, for the sample made of DeSolite DS-2015 polymer, cooling and subsequent heating rate 2 °C/min. Specimen size is 13.461 mm × 6.2 mm × 0.045 mm.

**Figure 3:**
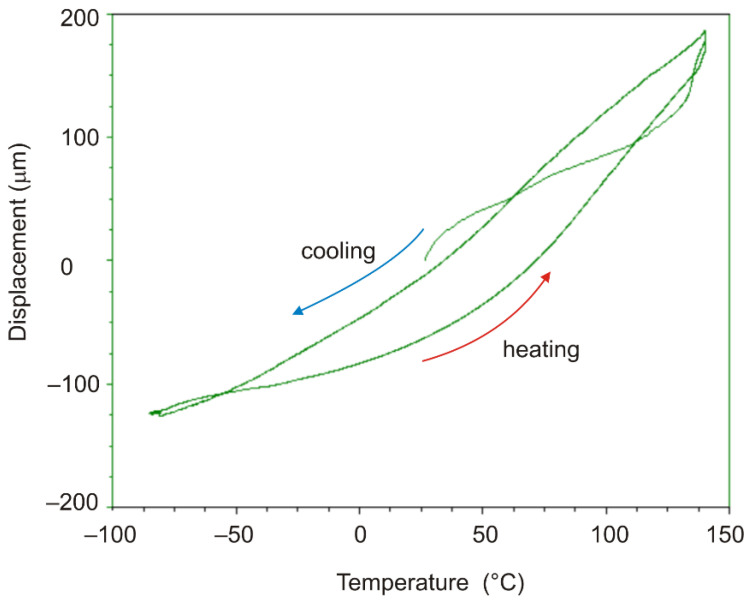
Characteristic temperature dependence of displacement, DeSolite DS-2015 polymer, cooling and subsequent heating rate 2 °C/min. Specimen size is 13.461 mm × 6.2 mm × 0.045 mm.

Throughout the experiment, changes in sample length (displacement), temperature, and force were recorded (Figure 2). Figure 3 shows the obtained dependences of displacement on temperature.

**Figure 4:**
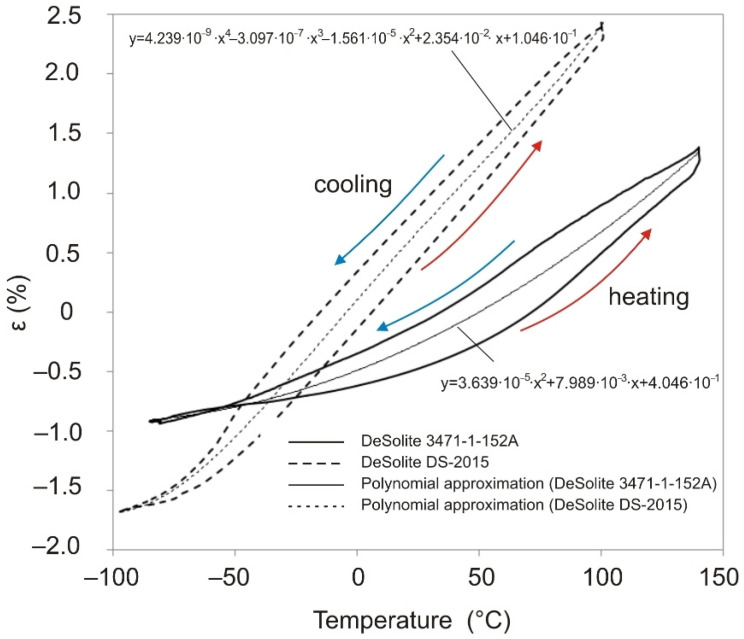
Temperature dependence of deformation for DeSolite 3471-1-152A (dashed line) and DeSolite DS-2015 (solid line) polymers, cooling/heating rate 2 °C/min. Polynomial approximation of the temperature dependence of the averaged strain values taken on heating and cooling.

The obtained dependences of displacements on time and temperature allow plotting the dependences of strain on temperature. These dependences turn out to be different when the sample is heated and cooled down. Averaging the displacement values obtained during heating and cooling of the sample allows us to obtain a dependence describing the strain response of the material when the temperature change rate is close to zero. This dependence can be approximated by a polynomial, shown for each material studied in Figure 4. This approach to estimating CTE is similar to the method given in the standard [21] and more accurately describes the material’s behavior than the tabular values provided by the manufacturer [14,15].

From the analysis of the results of the experiments shown in Figure 3 and Figure 4, it follows that the rate of temperature change can significantly affect the strain response of the polymer film; the strain response of the polymer to temperature when heated and cooled down at the same rate differs significantly. 

The experimental results obtained with film samples made of two UV-curable polymers, DeSolite 3471-1-152A and DeSolite DS-2015, provoked the desire to conduct a similar experiment for film samples made of a conventional polymer, EPO-TEK 330 epoxy resin (Epoxy technology inc., Billerica, MA, USA). The dimensions of the epoxy resin sample were length 14.071 mm, width 6.25 mm, thickness 0.245 mm.

**Figure 5:**
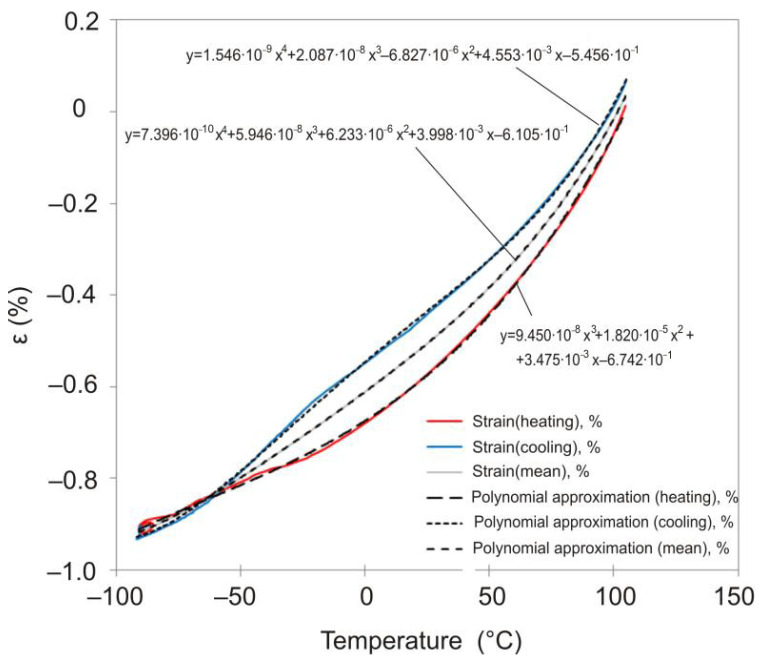
Temperature dependence of strain for EPO-TEK 330 polymer, cooling/heating rate 2 °C/min, sample thickness 245 µm. Approximations by a power polynomial of the temperature dependence of strain on heating, cooling, and their averaged values.

**Figure 6:**
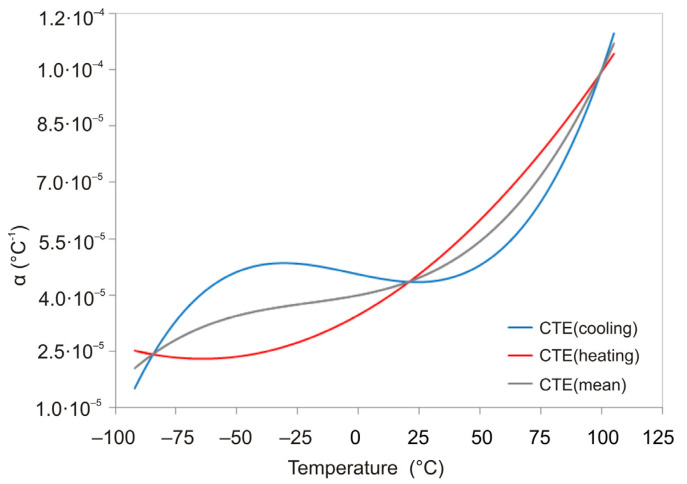
Temperature dependence of CTE of EPO-TEK 330 polymer during cooling (blue) and heating (red) at a rate of 2 °C/min, sample thickness 245 µm.

The graphical results shown in Figure 5 demonstrate the strain response of the EPO-TEK 330 epoxy resin film sample when the temperature changes at different rates. Figure 6 shows for this polymer the dependence of CTE on temperature obtained by differentiating the relation by temperature presented in Figure 5.

Thus, the experimental results obtained demonstrate that the coefficient of thermal expansion of the polymer may depend not only by the absolute value of the temperature but also on the rate of its change and the temperature history in general. The difference in the results obtained during heating and cooling down suggests that the polymer may accumulate residual strain under conditions of cyclic temperature change.

## 3. Results

### 3.1. Verification of the Hypotheses Stated

The area of the figure bounded by the graph of the dependence of the thermal expansion coefficient on temperature is known to be nothing more than the temperature strain. The area bounded by the cooling down and heating curves corresponds to the residual temperature strain accumulated in one step of the cycle. Thus, if the heating and cooling of the polymer film are repeated cyclically, the mismatch of the plots corresponding to these steps indicates the accumulation of residual strains at each step of the cycle.

**Figure 7:**
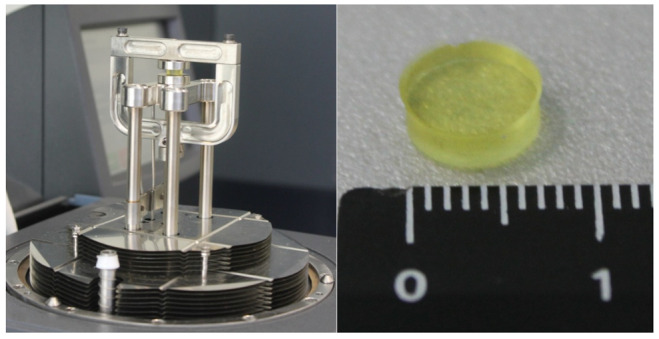
Cylindrical shape specimen and tooling for its thermal cycling tests.

**Figure 8:**
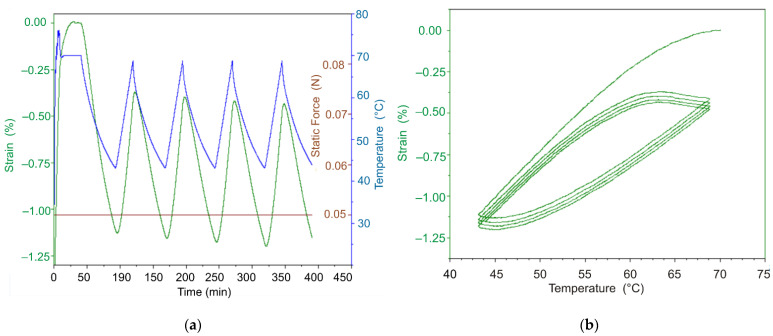
Evolution of temperature strain of the cylindrical sample under thermocycling conditions (**a**) and strain-temperature diagram (**b**). Thermal expansion of the cylindrical specimen under the controlled force of 0.05 N, cyclically varying temperature in the range 43–68 °C, at a heating rate of 2 °C/min, and with natural cooling. The strain is counted from 40 min, corresponding to the beginning of the thermocycling and temperature 70 °C.

To verify the discovered effect on more massive objects, the following experiment was carried out. The cylindrical DeSolite 3471-1-152A polymer sample with 7.36 mm diameter and 2.769 mm height fixed in suitable clamps (Figure 7) was heated and then cooled down following the temperature change program shown in Figure 8a. The sample was kept in the gripper under constant compressive load F = 0.05 N. In the study process, the change of sample length (displacement), temperature, and force were recorded. The temperature was changed in the range of 43–70 °C, the material of the sample in the specified interval is in a high-elastic state. Figure 8b shows the obtained dependence of temperature strain of the cylindrical sample on the temperature during four cycles of heating–cooling. At the starting point of the cyclic process (40th minute in Figure 8a) the temperature of the sample is 70 °C.

**Figure 9:**
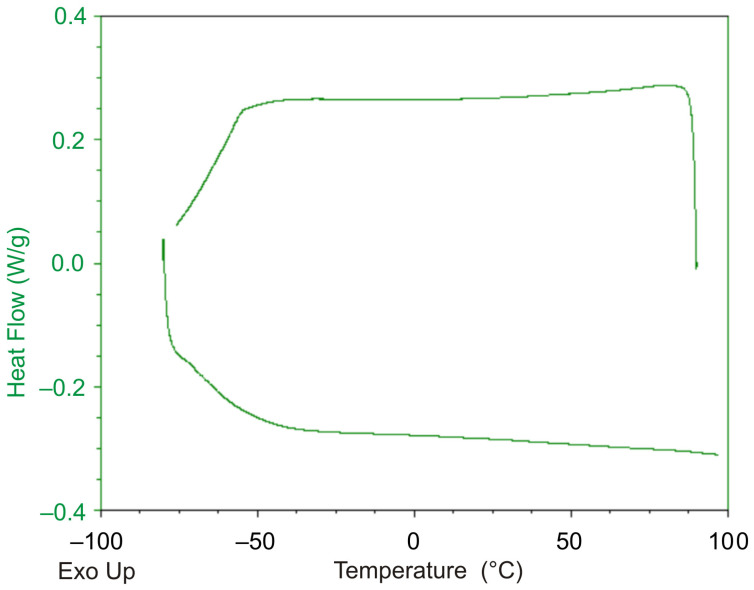
Dependence of heat flow on temperature recorded for DeSolite 3471-1-152A polymer by DSC TA Instruments Q2000.

The results shown in Figure 8a, b indicate that with each round of the thermal cycle, the temperature strain accumulates in the sample. The observed situation cannot be explained by the effects related to glass transition or changes in the compliance properties, since the glass transition temperature for this polymer lies in the region of negative temperatures, and the stiffness of the specimen in the temperature interval under study practically does not change (Figure 1). The experiment on a differential scanning calorimeter (DSC) Q2000 of TA Instruments (TA Instruments Inc., New Castle, DE, USA) showed (Figure 9) that no exo- or endothermic reactions related to crystallization or melting processes, physical aging, or other thermodynamic processes occur in the polymer in temperature interval of 40–70 °C.

Thus, the experiments conducted confirm the hypothesis that conditions for the accumulation of temperature strains can be achieved in polymers under conditions of cyclic temperature changes.

**Figure 10:**
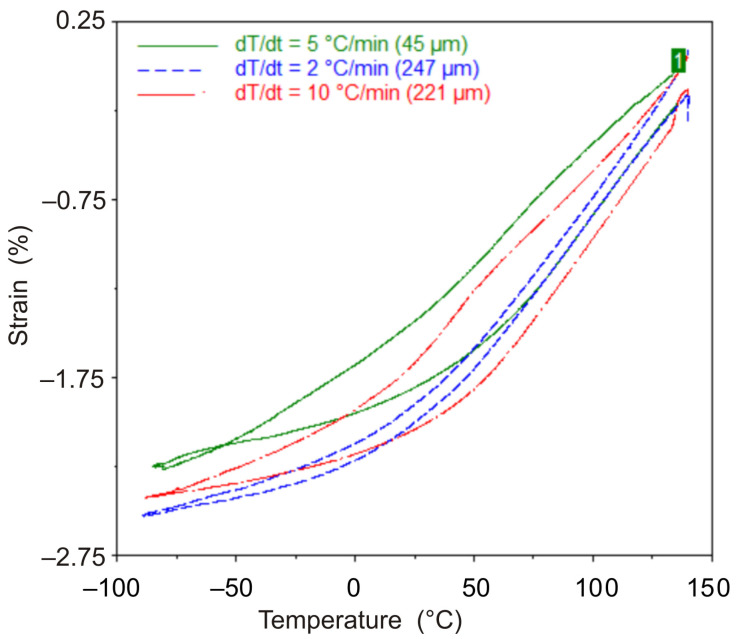
Dependence of strain on temperature for DeSolite DS-2015 polymer specimens with different thicknesses and cooling/heating rates: 45 µm, 5 °C/min (green); 221 µm, 10 °C/min (red); 247 µm, 2 °C/min (blue).

**Figure 11:**
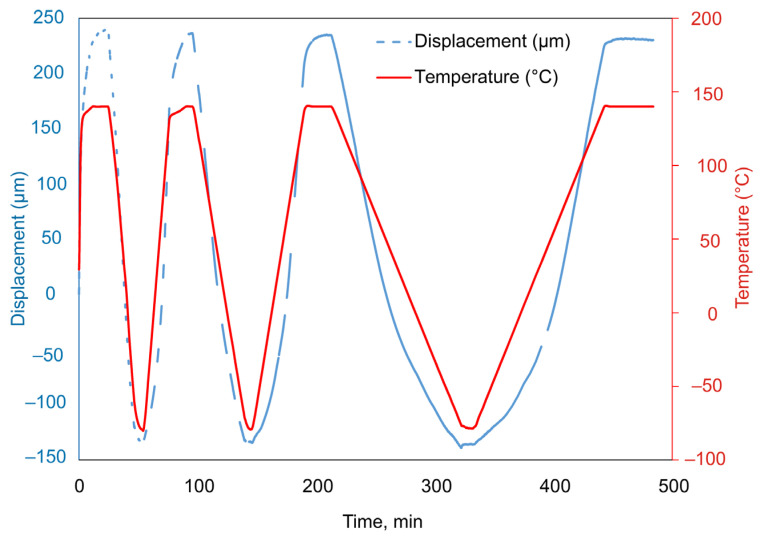
Time dependence of the parameters recorded in experiment, for the DeSolite DS-2015 polymer specimen.

**Figure 12:**
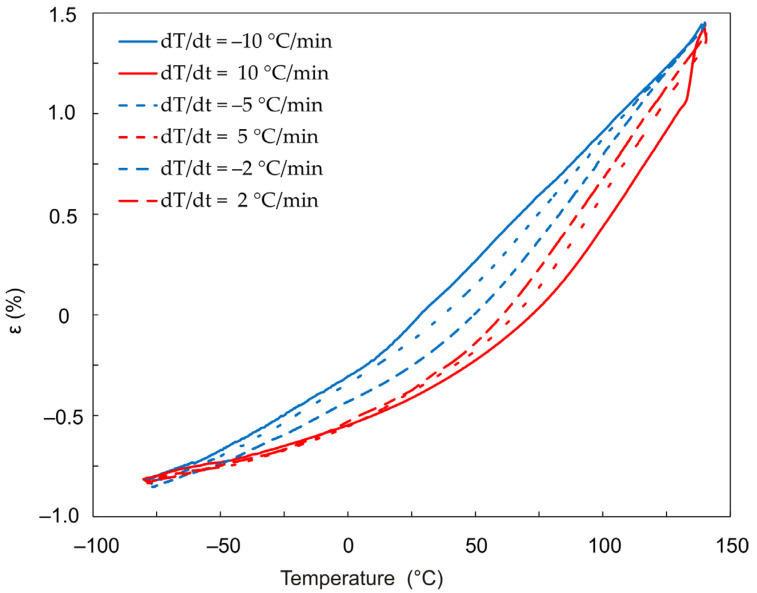
Temperature dependence of strain for 42 µm DeSolite DS-2015 polymer specimen with different cooling/heating rates: 10 °C/min (heating—solid red line/cooling—solid blue line); 5 °C/min (red/blue dash line); 2 °C/min (red/blue dash-dot line).

We performed two series of similar experiments using film samples. In the first series, film-shaped samples of different thicknesses were subjected to heating and cooling following the program shown in Figure 2. Different heating/cooling rates were set (Figure 10). In the second case, a 42 μm-thick sample was heated and cooled down at different rates (Figure 11 and Figure 12). Figure 10 and Figure 12 show the results obtained. Analysis of the obtained data allows us to conclude that the higher the rate of temperature change, the more the curves corresponding to heating and cooling down are separated along the abscissa axis (Figure 12). Accordingly, the lower the rate of temperature change, the closer to each other the parameters recorded during heating and cooling down.

**Figure 13:**
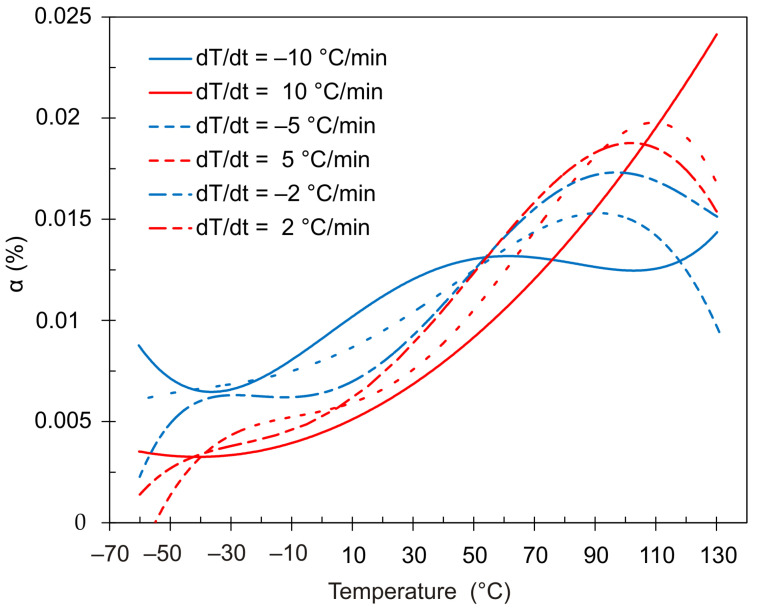
Dependence of the thermal expansion coefficient α(T) for a 42 µm thick film sample made of DeSolite DS-2015 polymer, with different cooling/heating rates: 10 °C/min (heating—solid red line/cooling—solid blue line); 5 °C/min (red/blue dash line); 2 °C/min (red/blue dash-dot line).

Figure 13 shows plots of the CTE change as a function of temperature, obtained at 3 different rates of temperature change. The curves represent the data obtained by differentiating by temperature the dependences presented in Figure 12.

**Figure 14:**
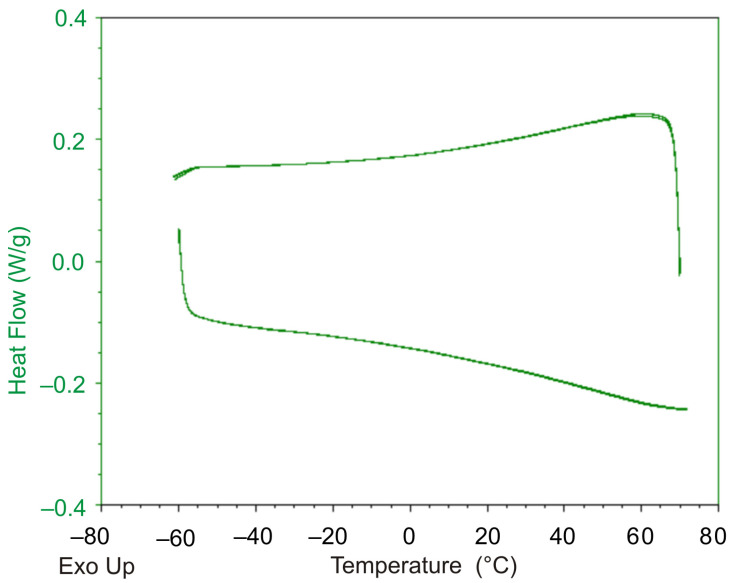
Dependence of heat flow on temperature recorded for DeSolite DS-2015 polymer (differential scanning calorimeter data).

During the study of film samples of DeSolite DS-2015 polymer on a differential scanning calorimeter, we confirmed that no thermodynamic effects associated with phase transitions in the polymer were observed (Figure 14).

The described phenomena contribute significantly to the pattern of thermomechanical processes taking shape in thin polymer films or fibers. In larger polymer products, the effects described above may lead to the development of residual stress fields if significant temperature gradients occur or unsteady thermal processes are implemented. Repeated heating and cooling down during the use of polymer products can also cause the accumulation of residual strains. A detailed analysis of such thermomechanical processes in volumetric structures made of glassy polymers is possible based on numerical simulation, which should account for experimentally established regularities.

### 3.2. Verification of Obtained Results

To verify the obtained regularities, we conducted a series of experiments to compare the CTE value measured by two methods: described above using DMA and the traditional one using a TA DL 802 horizontal dilatometer. Given the specifics of the dilatometer, the temperature range had to be significantly limited, and the temperature range corresponding to the relaxation transition must be excluded to perform the study. We select EPO-TEK 330 epoxy resin as the most suitable material from which samples in the form of 3.11 mm × 2.03 mm × 11.94 mm bars for tests in the dilatometer and 170 µm thick films for tests on the Q800 DMA. Table 2 shows the material properties presented by the manufacturer.

**Figure 15:**
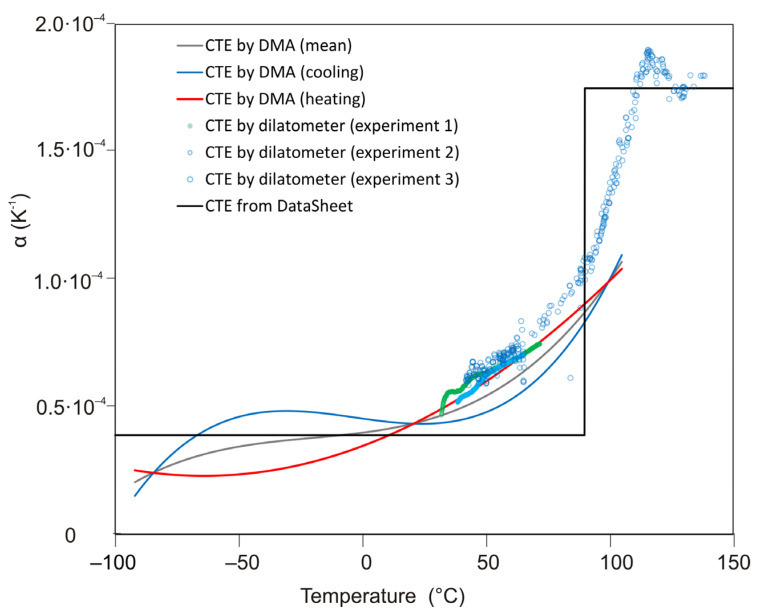
Dependence of the thermal expansion coefficient α(T) for EPO-TEK 330 polymer, measured by DMA, dilatometer and from manufacturer datasheet. Cooling/heating rates are 2 °C/min (except experiment 3, where the cooling rate is 1 °C/min).

Figure 5 shows the results of measurements on the dynamic mechanical analyzer. The experimental dependences of the strain on temperature were approximated by power functions. By differentiating these dependencies by temperature, the corresponding CTE dependencies were obtained (Figure 6). The temperature dependence of CTE was also obtained experimentally using a dilatometer TA Instruments DIL802 (TA Instruments inc., New Castle, DE, USA). Figure 15 compares these data with the results obtained using DMA. The same graph shows the CTE values given by the manufacturer of EPO-TEK 330 epoxy resin. It follows from the comparison results: the experimental data obtained on the dilatometer satisfactorily coincide with the results obtained by DMA in the temperature ranges under consideration; the average value of CTE according to DMA in the temperature range below the glass transition temperature is close to the value of CTE according to the manufacturer. We believe that these results indicate the applicability of our developed technique for measuring the CTE as a function of temperature and its rate for polymeric materials.

## 4. Conclusions

This paper proposes an approach to studying the strain response of film samples of glassy polymer materials to temperature effects in different modes. The advantages of the developed approach are largely determined by the fact that thin films of material are used as samples, in which it is possible to form a sufficiently uniform temperature field in a wide range of temperature change rates. A dynamic mechanical analyzer DMA TA Q800 was used to implement this approach.

The results of the experiments carried out following the developed approach have made it possible to establish the following:

- The linear thermal expansion coefficient significantly depends not only on the temperature but also on the rate of its change.
- The strain response of the polymer to temperature during heating and cooling down, with the same absolute values of the temperature change rate, differs significantly; this difference becomes stronger as the temperature change rate increases.
- During cyclic temperature change of the material, a residual strain is generated due to the difference in the strain response of the polymer to temperature change in the process of heating and cooling down.

The established dependencies of the CTE on the temperature and the rate of its change can be used in the mathematical modeling of thermomechanical processes arising during the operation of products made of glassy polymeric materials.

## Figures and Tables

**Table 1:** CTE of DeSolite polymers, according to the manufacturer [14,15].

Polymer Grade	Glass Transition Range (DMA), °C at E’, 100 MPa	Glass Transition Range (DMA), °C at E’, 1000 MPa	Coefficient of Expansion (TMA), 500 μm Films in the Glassy Region (×10^−6^), °C^−1^	Coefficient of Expansion (TMA), 500 μm Films in the Rubbery Region (×10^−6^), °C^−1^
13471-1-152A	−54	−65	<100	660
DS-2015	80	40	38	196

**Table 2:** Coefficients of thermal expansion, according to the manufacturer [22].

Polymer Grade	CTE in the Glassy Region (×10−6), °C^−1^	CTE in the Rubbery Region (×10−6), °C^−1^
EPO-TEK 330	−54	−65
